# Reduced virulence of a pseudorabies virus isolate from wild boar origin in domestic pigs correlates with hampered visceral spread and age-dependent reduced neuroinvasive capacity

**DOI:** 10.1080/21505594.2017.1368941

**Published:** 2017-10-04

**Authors:** Sara Verpoest, Valerie Redant, Ann Brigitte Cay, Herman Favoreel, Nick De Regge

**Affiliations:** a Operational Direction Viral Diseases, CODA-CERVA, Ukkel, Belgium; b Department of Virology, Immunology and Parasitology, Faculty of Veterinary Medicine, Ghent University, Merelbeke, Belgium

**Keywords:** neuroinvasion, olfactory bulb, pathogenesis, pseudorabies virus, virulence, wild boar

## Abstract

Morbidity and mortality associated with pseudorabies virus (PRV) infection are dependent on the age of the pig and the virulence of the strain. PRV strains circulating in wild boar are considered to be low virulent, but no mechanistic explanation for their reduced virulence is available. Here infection of 2- and 15-week-old domestic pigs with the PRV wild boar strain BEL24043 did not induce clinical symptoms in 15-week-old pigs, but resulted in important neurological and respiratory disease in 2-week-old piglets. A detailed study of the (neuro) pathogenesis and associated cytokine mRNA expression showed that the reduced virulence of the wild boar strain, compared to what was previously reported for the virulent domestic NIA3 strain, is due to a severely hampered spread to visceral organs in pigs of both age categories and to an efficient suppression of viral replication at primary replication sites of 15-week-old pigs and to a lesser extent in those of 2-week-old piglets. The age-dependent difference in induced symptoms seems to be due to an immature development state of the immune and/or nervous system in 2-week-old pigs. An extensive viral replication associated with a robust expression of cytokine-related mRNA was found in the olfactory bulb of 2-week-old piglets, correlating with observed neurological disease. Neuroinvasion also occurred via the trigeminal route in 2-week-old pigs, but viral replication was efficiently suppressed in the trigeminal ganglion in the presence of a moderate induction of cytokine-related mRNA. Viral replication in the peripheral and central nervous system of 15-week-old pigs was limited and efficiently suppressed.

## Introduction

Pseudorabies virus (PRV), also called suid herpesvirus 1 or Aujeszky's disease virus, is the causative agent of the economically important Aujeszky's disease in swine. PRV belongs to the family *Herpesviridae,* subfamily *Alphaherpesvirinae,* genus *Varicellovirus*. Members of the family *Suidae* are the only natural hosts and reservoirs of the virus. Infection of its natural host is characterized by respiratory, reproductive and neurological symptoms. The severity of the induced symptoms depends on the virulence of the strain and the age of the pig.^1^^,^^2^

Differences in virulence between strains circulating in domestic pigs have been well documented. Whereas Aujeszky's disease occurred only sporadically before the 1960s, more severe outbreaks of PRV in pigs associated with a change in clinical symptoms were observed from the 1960–70s onwards, indicative of the emergence of more virulent PRV strains.^3^^,^^4^ Upper respiratory tract problems accompanied by severe general symptoms such as fever and anorexia became more prominent. The increase in invasive character at the level of the respiratory tract allowed the virus to reach nerves more effectively, to increase viremia and to replicate to high levels in internal organs, including reproductive organs. Accordingly, an increase in neurological symptoms and mortality was observed in piglets, and mortality and abortion also appeared in older animals.^4^ As a result of the increased economic impact of the virus, large-scale vaccination programmes were set up which led to the eradication of the virus in many countries.^2^

PRV remains however present in the wild boar population and poses a risk for reintroduction of the virus in currently unprotected domestic swine. Strains circulating in wild boar are generally assumed to be less virulent than isolates that circulated in domestic swine.^5^^,^^6^ Recently, we showed that differences in virulence also exist between wild boar isolates. While two Belgian wild boar strains were attenuated in adult pigs (BEL24043 and BEL20075), only one induced important respiratory and neurological disease in 2-week-old piglets (BEL24043).^7^ Overall, cell biological and molecular explanations for the observed differences in virulence between PRV strains, and particularly wild boar strains, are lacking.

Age and immune status of the pig are also important determinants in the outcome of a PRV infection. Morbidity and mortality associated with PRV infection are higher in younger pigs, and typically associated with symptoms of the CNS. Older swine mostly exhibit symptoms of respiratory and reproductive disease.^2^ Recently, the results of an in vivo infection study of 2- and 15-week old pigs with the virulent PRV NIA3 strain suggested that age-dependent differences in PRV induced clinical signs are due to enhanced viral replication and associated immunopathology in immature trigeminal ganglion and central nervous system neurons of 2-week old pigs and confirmed previous findings that neurological disease seems related with extensive viral replication and an associated immune response in the olfactory bulb.^8-11^

In this report, we present a study of the pathogenesis and associated cytokine mRNA expression in 2- and 15-week-old domestic pigs after infection with the previously characterized wild boar PRV isolate BEL24043.^7^ In addition, we compare and contrast these results to our previous study with the virulent domestic NIA3 strain^8^ and from this suggest mechanistic explanations for the age-dependence of infection and the overall reduced virulence of the BEL24043 strain.

## Results

### Clinical symptoms

Inoculation of 2-week-old piglets with the wild boar strain induced important respiratory and neurological disease. Starting from 4 days p.i., the piglets showed a slight increase in body temperature (±0.5°C), a reduced appetite and were lethargic. Between 5 and 9 days p.i. all piglets experienced general (vomiting, diarrhea), respiratory (heavy breathing) and/or neurological (trembling, scratching) symptoms. One piglet (W11) had to be euthanized at 5 days p.i. based on ethical grounds instead of its foreseen euthanasia at 21 days p.i. The general condition of the remaining piglets improved after 9 days p.i.

In contrast, no clinical symptoms were observed in 15-week-old pigs inoculated with the wild boar isolate. The general condition of the animals, food consumption, mobility and rectal body temperature remained apparently unaffected throughout the experiment.

### Pseudorabies virus DNA detection in the nervous system and visceral organs

**Figure 1.**
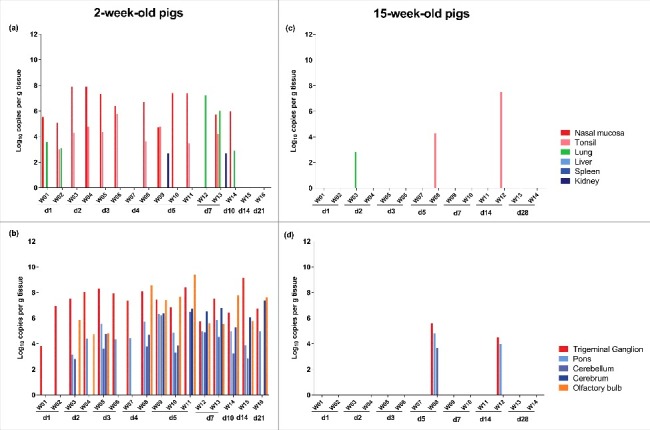
Distribution of viral DNA in different tissues after infection of domestic pigs with the wild boar PRV strain BEL24043. Several tissues were collected from domestic pigs of 2- and 15-week-old at different time points after intranasal inoculation with the wild boar PRV strain BEL24043 and tested by qPCR directed against glycoprotein gB. Viral DNA concentrations at different primary and secondary sites of replication for 2- and 15-week-old pigs (a and c, respectively) and for peripheral and central nervous system tissues for 2- and 15-week-old pigs (b and d, respectively) are shown. Different pigs infected with the wild boar strain are indicated by W01 to W16 and d1 to d28 indicate the day post infection at which the pigs were euthanized.

Analysis of the presence of viral DNA by qPCR in the different tissues collected at the day of euthanasia indicated that the wild boar strain quickly invaded the PNS and CNS of 2-week-old piglets after intranasal inoculation (Fig. 1).

From 24 hours p.i. onwards, viral DNA could be detected in the nasal mucosa and tonsils of most piglets till 7 days p.i. No positive tonsils were found from 10 days p.i. onwards and nasal mucosa were viral DNA negative from 14 days p.i.

At 24 hours p.i., virus had also already reached the TG in both piglets, indicating efficient neuroinvasion. At 48 hours p.i., virus had also invaded the CNS where it was detected in the pons, cerebellum and/or olfactory bulb. Both at 3 and 4 days p.i., each time one piglet was positive for all brain tissues tested while the other was only positive in the TG and pons. Between 5 and 21 days p.i. brain tissues of all piglets remained positive for viral DNA.

In contrast to the efficient spread to the nervous system, only a very limited dissemination to the visceral organs was observed after inoculation of 2-week-old pigs with the wild boar isolate, which is in stark contrast with what has been described before for the domestic NIA3 PRV strain (Fig. S1).^8^ Besides the initial detection of viral DNA in the lung at 24 hours p.i., positive lung samples were only found at 7 and 10 days p.i. Only renal tissue of two piglets, one euthanized at 5 days p.i. and another at 7 days p.i., was found positive. No viral DNA was detected in liver or spleen samples.

In 15-week-old pigs, only a very limited replication at the primary inoculation site and dissemination toward the nervous system and visceral organs was found after inoculation with the wild boar strain (Fig. 1). Only in three animals, tissues positive for viral DNA were found at the moment of euthanasia. One animal euthanized at 2 days p.i. was positive in lung tissue. Two other sows (W08 and W12) were positive for viral DNA in tonsils at 5 and 14 days p.i., respectively. Interestingly, the same two sows were also positive in the TG and pons. The viral DNA load in the TG of these 15-week-old sows (approximately 10^5^ copies/g) was however substantially lower than the viral DNA load found in TG of 2-week-old piglets (between 10^7^–10^8^ copies/g). Despite the detection of viral DNA in tonsils, PNS and CNS samples of these 15-week-old pigs (W08 and W12), no clinical symptoms were observed in these animals.

### Viral and cytokine gene expression at distinctive sites involved in neuropathogenesis

#### Nasal mucosa

**Figure 2.**
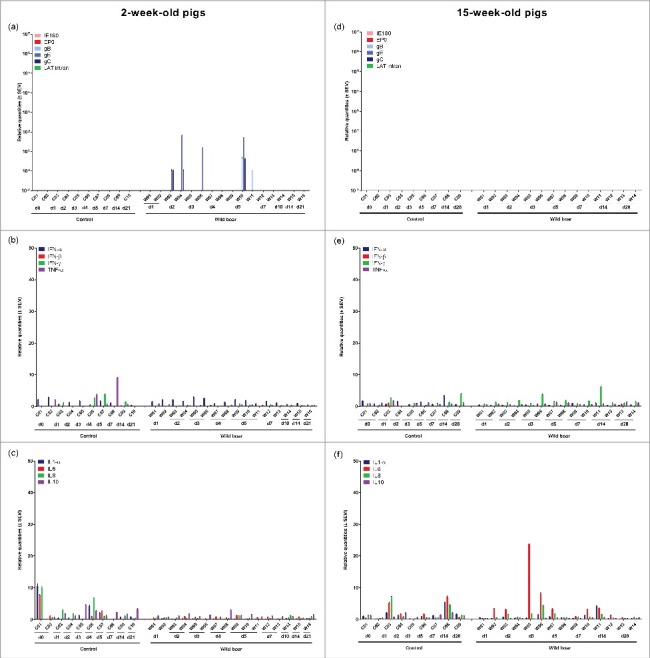
Nasal mucosa. Domestic pigs of 2- and 15-weeks old were intranasally inoculated with the wild boar PRV strain BEL24043 and euthanized at different time points post infection. mRNA expression of several PRV genes in the nasal mucosa was studied by RT-qPCR in 2- and 15-week-old pigs (a and d, respectively). Viral gene levels were expressed relative to the lowest positive sample for each age category. Furthermore, cytokine-related mRNA expression in de nasal mucosa was tested by RT-qPCR in 2- and 15-week-old pigs (b, c and e, f, respectively). Individual cytokine levels for all animals are expressed relative to the average cytokine expression in the control group (separately for 2- and 15- week old animals). Different pigs infected with the wild boar strain are indicated by W01 to W16. Control pigs are indicated by C01 to C10. d1 to d28 indicate the day post infection at which the pigs were euthanized.

Viral mRNA expression in the nasal mucosa of 2-week-old piglets was low and limited to only a few piglets after infection with the wild boar strain (Fig. 2a). Expression of IE180, EP0 and LAT intron was never detected. In 15-week-old pigs, no viral mRNA was detected at all, correlating with the absence of detectable viral DNA in the nasal mucosa (Fig. 2d).

No clear upregulation of cytokine mRNA expression was observed in the nasal mucosa after inoculation of 2-week-old piglets with the wild boar strain (Fig. 2b and c). Only in some 15-week-old pigs, a modest increase in cytokine mRNA was found for IL-6 (Fig. 2e and f). However, in these particular pigs, no viral DNA or mRNA was detected.

#### Tonsils

**Figure 3.**
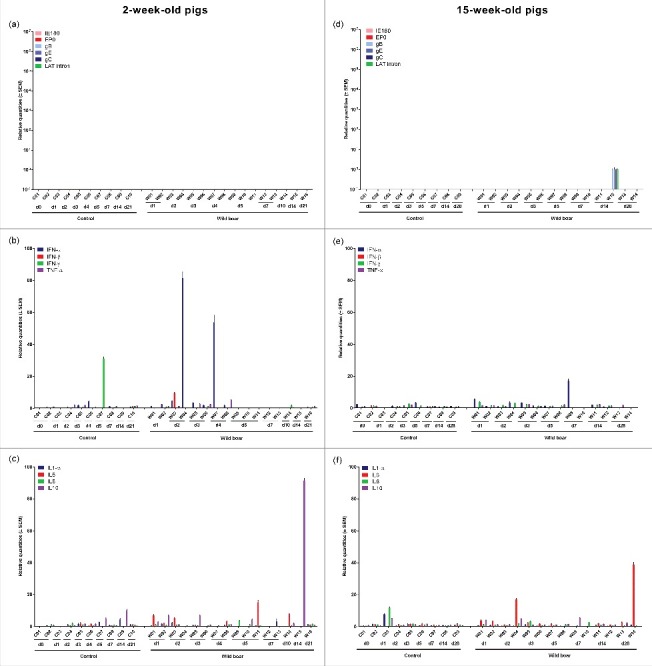
Tonsils. Domestic pigs of 2- and 15-weeks old were intranasally inoculated with the wild boar PRV strain BEL24043 and euthanized at different time points post infection. mRNA expression of several PRV genes in the tonsils was studied by RT-qPCR in 2- and 15-week-old pigs (a and d, respectively). Viral gene levels were expressed relative to the lowest positive sample for each age category. Furthermore, cytokine-related mRNA expression in de nasal mucosa was tested by RT-qPCR in 2- and 15-week-old pigs (b, c and e, f, respectively). Individual cytokine levels for all animals are expressed relative to the average cytokine expression in the control group (separately for 2- and 15- week old animals). Different pigs infected with the wild boar strain are indicated by W01 to W16. Control pigs are indicated by C01 to C10. d1 to d28 indicate the day post infection at which the pigs were euthanized.

Despite the detection of viral DNA in tonsils of most 2-week-old pigs, viral mRNA expression in these tonsils was never detected (Fig. 3a). Viral mRNA was only found in one (W12, euthanized at 14 days p.i) 15-week-old pig (Fig. 3d). The latter was one of two 15-week-old pigs in which also viral DNA was detected in tonsils.

No consistent increase in cytokine mRNA was found. Only some 2-week-old pigs showed an upregulation of IFN-α (maximum of 80-fold) and IL-10 mRNA (maximum of 90-fold) (Fig. 3b and c), and two 15-week-old pigs showed an increase in IL-10 mRNA expression (maximum 40-fold) (Fig. 3f).

Since tonsils were also collected in our earlier study on pigs infected with the virulent domestic NIA3 strain^8^ but had not been analyzed for viral or cytokine mRNA expression, these samples were analyzed in the current study. Only very limited viral mRNA expression was found in tonsils of 2-week-old piglets during the first 4 days after NIA3 infection (end of the experiment, Fig. S2), despite the presence of substantial amounts of viral DNA. Only 3 piglets were found positive for maximal one viral mRNA. In contrast, expression of viral glycoprotein mRNA was detected in tonsils of most 15-week-old pigs between 3 and 7 days p.i. The latter seemed to be associated with a moderate increase in IL-6 (maximum 11-fold) and IL-10 (maximum 14-fold) transcripts in most 15-week-old pigs.

#### Trigeminal ganglion

**Figure 4.**
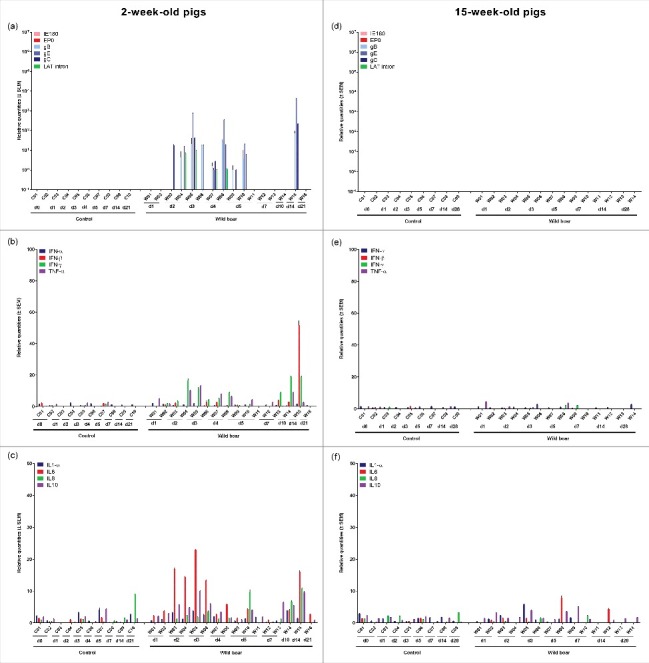
Trigeminal ganglion. Domestic pigs of 2- and 15-weeks old were intranasally inoculated with the wild boar PRV strain BEL24043 and euthanized at different time points post infection. mRNA expression of several PRV genes in the trigeminal ganglion was studied by RT-qPCR in 2- and 15-week-old pigs (a and d, respectively). Viral gene levels were expressed relative to the lowest positive sample for each age category. Furthermore, cytokine-related mRNA expression in de trigeminal ganglion was tested by RT-qPCR in 2- and 15-week-old pigs (b, c and e, f, respectively). Individual cytokine levels for all animals are expressed relative to the average cytokine expression in the control group (separately for 2- and 15- week old animals). Different pigs infected with the wild boar strain are indicated by W01 to W16. Control pigs are indicated by C01 to C10. d1 to d28 indicate the day post infection at which the pigs were euthanized.

In 2-week-old pigs, viral mRNA was first detected in the TG at 48 hours p.i., i.e. 24 hours after PRV DNA detection. Viral mRNA was detected in all piglets euthanized between 2 and 5 days p.i. (Fig. 4a) but its expression was not always consistent. Expression of mRNA of all late genes studied and LAT intron was only found in some piglets and IE180 and EP0 mRNA was never detected. Between 6 and 21 days p.i. viral gene expression was no longer detected in the TG, except for the piglet euthanized at 14 days p.i. In contrast, no viral mRNA was detected at any time point in the TG of 15-week-old pigs (Fig. 4d).

After inoculation of 2-week-old piglets, a moderate upregulation of cytokine related mRNA was observed in those animals that showed detectable viral mRNA expression. The increase in cytokine mRNA expression occurred simultaneously with the appearance of viral mRNA and an increase in expression of IL6 (maximum increase of 23 fold), IFN-γ (maximum increase of 19 fold), TNF-α (maximum increase of 13 fold) and IL10 (maximum increase of 11 fold) was observed (Fig. 4b and c). No clear upregulation of cytokine-related mRNA was observed in the TG of 15-week-old pigs (Fig. 4e and f).

#### Brainstem

**Figure 5.**
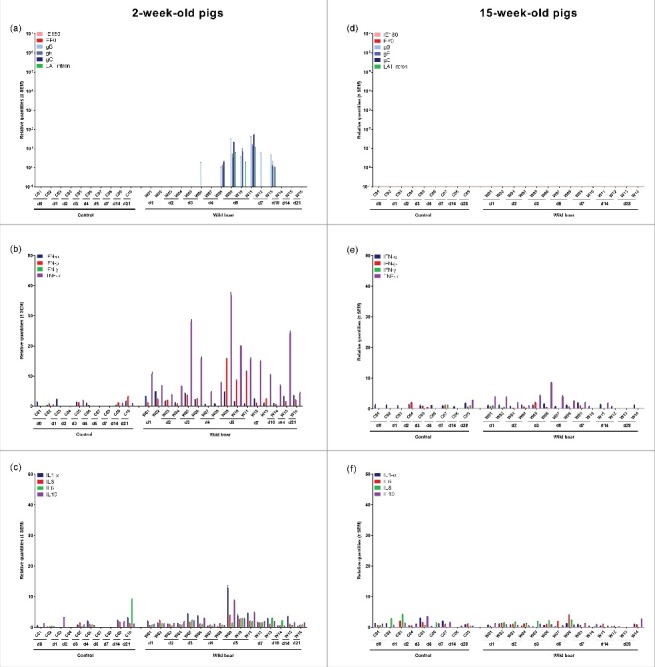
Pons. Domestic pigs of 2- and 15-weeks old were intranasally inoculated with the wild boar PRV strain BEL24043 and euthanized at different time points post infection. mRNA expression of several PRV genes in the pons was studied by RT-qPCR in 2- and 15-week-old pigs (a and d, respectively). Viral gene levels were expressed relative to the lowest positive sample for each age category. Furthermore, cytokine-related mRNA expression in de pons was tested by RT-qPCR in 2- and 15-week-old pigs (b, c and e, f, respectively). Individual cytokine levels for all animals are expressed relative to the average cytokine expression in the control group (separately for 2- and 15- week old animals). Different pigs infected with the wild boar strain are indicated by W01 to W16. Control pigs are indicated by C01 to C10. d1 to d28 indicate the day post infection at which the pigs were euthanized.

Late viral glycoprotein mRNA and sometimes LAT intron RNA was detected in the pons of 2-week-old piglets for one of both piglets at 3 and 4 days p.i. and for all piglets between 5 and 7 days p.i. (Fig. 5a). On the other hand, no viral mRNA expression was detected at all in the brainstem of 15-week-old pigs (Fig. 5d).

In 2-week-old piglets, detection of viral mRNA was accompanied by an upregulated TNF-α mRNA expression (with a maximum increase of 28 fold at 6 days p.i.) and an increase at 5 and 6 days p.i. of IFN-β (maximum increase of 12 fold). A more limited increase in IL1-α (maximum increase of 8 fold) and IL10 (maximum increase of 6 fold) mRNA expression was also detected (Fig. 5b and c). On the other hand, no clear upregulation of cytokine-related mRNA was observed in 15-week-old pigs (Fig. 5e and f).

#### Olfactory bulb

**Figure 6.**
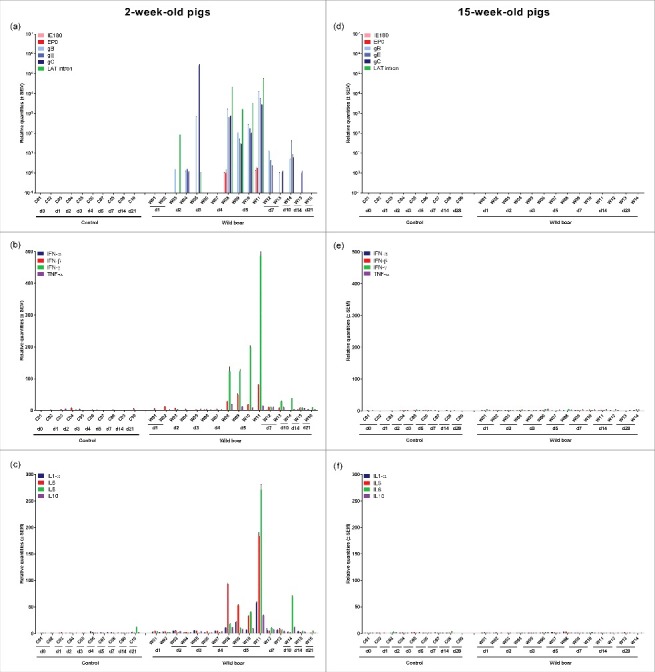
Olfactory bulb. Domestic pigs of 2- and 15-weeks old were intranasally inoculated with the wild boar PRV strain BEL24043 and euthanized at different time points post infection. mRNA expression of several PRV genes in the olfactory bulb was studied by RT-qPCR in 2- and 15-week-old pigs (a and d, respectively). Viral gene levels were expressed relative to the lowest positive sample for each age category. Furthermore, cytokine-related mRNA expression in de olfactory bulb was tested by RT-qPCR in 2- and 15-week-old pigs (b, c and e, f, respectively). Individual cytokine levels for all animals are expressed relative to the average cytokine expression in the control group (separately for 2- and 15- week old animals). Different pigs infected with the wild boar strain are indicated by W01 to W16. Control pigs are indicated by C01 to C10. d1 to d28 indicate the day post infection at which the pigs were euthanized.

After inoculation of 2-week-old pigs, mRNA of viral glycoproteins and the LAT intron was already detected in the olfactory bulb at 2 and 3 days p.i. in those animals that were also found positive for viral DNA (Fig. 6a). Starting from 4 days p.i., viral replication increased to a higher level than detected in the TG and brainstem and became more consistent with detection of mRNA of all late viral glycoproteins studied. In two piglets (W08 and W11), IE180 and EP0 mRNA was also detected. From 7 days p.i. onwards, viral mRNA levels started to decrease and mRNA expression was limited to one or more glycoproteins. Consistent with the absence of detectable viral DNA in the olfactory bulb of 15-week-old pigs, no viral mRNA expression could be observed in pigs of this age (Fig. 6d).

One to two days after the onset of viral replication in the olfactory bulb of 2-week-old piglets, a robust increase in cytokine-related mRNA expression was detected (Fig. 6b and c). A strong upregulation of IFN-β (maximum average increase of 35 fold), IFN-γ (maximum average increase of 161 fold), TNF-α (maximum average increase of 12 fold), IL1-α (maximum average increase of 15 fold), IL6 (maximum average increase of 51 fold), IL8 (maximum average increase of 25 fold) and IL10 (maximum average increase of 8 fold) was observed in piglets euthanized at 4 and 5 days p.i. An important observation was made for piglet W11 that experienced severe neurological symptoms and had to be euthanized at 5 days p.i. based on ethical grounds. Cytokine related mRNA expression in this piglet was even more pronounced compared to the expression in the other animals euthanized at 4 and 5 days p.i., with a massive increase in IFN-γ (increase of 487 fold), IFN-β (increase of 81 fold), TNF-α (increase of 14 fold), IL1-α (increase of 58 fold), IL6 (increase of 184 fold), IL8 (increase of 271 fold) and IL10 (increase of 34 fold). From 7 day p.i. onwards, cytokine-related mRNA concentrations abruptly started to decrease again, returning to baseline levels at 14 and 21 days p.i. Again, no changes in cytokine-related mRNA expression were observed in the olfactory bulb of 15-week-old pigs (Fig. 6e and f).

### Detection of PRV-specific serum antibodies by ELISA

Since almost no viral DNA or mRNA positive samples were detected in 15-week-old pigs, we verified if infection with the wild boar isolate had nevertheless taken place by testing the presence of PRV specific antibodies in blood samples collected at the day of euthanasia via an ELISA directed against glycoprotein gB. Logically, no PRV-specific antibodies were detected in pigs euthanized between 1 and 5 days p.i. However, in the pigs euthanized at 7, 14 and 28 days p.i., each time one of both animals (W09, W12, W14) had seroconverted, indicating that infection had taken place. For one of these ELISA-positive animals (W12), viral DNA was detected in the tonsils, TG and pons. For the other two positive animals (W09, W14), no viral DNA or mRNA was detected at the day of euthanasia in any tissue.

## Discussion

Wild boar PRV strains are considered to be attenuated compared to strains circulating in domestic pigs.^5^^,^^6^ This was described before for the wild boar strain BEL24043 and is confirmed here.^7^ This strain did not induce clinical symptoms after infection of 15-week-old pigs, but interestingly, infection of 2-week-old piglets resulted in important respiratory and neurological disease. The severity of clinical symptoms and mortality were however clearly lower compared to those observed after infection of 2- and 15-week old pigs with the virulent NIA3 strain.^8^ To find potential explanations for these age- and strain-dependent differences in induced clinical disease, we performed a detailed analysis of viral DNA and mRNA expression in several tissues involved in neuropathogenesis after infection with the wild boar isolate, analogous to a previous study using the virulent domestic PRV NIA3 strain.^8^ This allowed to compare data of wild boar and domestic PRV infection. It was also evaluated whether a correlation could be found between the neuropathogenesis and the induction of cytokine mRNA expression.

A first important difference in pathogenesis observed in both 2- and 15-week-old pigs after infection with the wild boar strain on the one hand and the NIA3 strain on the other was the difference in viral dissemination to visceral organs (Figs. S1 and S2). Where viral DNA was detected rather quickly (between 2 and 4 days in young piglets and between 3 and 7 days for 15-week-old pigs) in the kidney, spleen and liver upon infection with NIA3,^8^ viral DNA was only detected in renal tissue of two 2-week-old piglets after infection with the wild boar strain. This could indicate that the wild boar strain may be less capable of reaching lymph and blood vessels. First potential explanations could be that the wild boar strain is hindered in its mobility through the mucus layer to reach the epithelial cells^12^ or has a reduced capacity to replicate in epithelial cells at the primary sites of infection. This might hold true for 15-week-old pigs since neither viral DNA nor viral mRNA was detected in the nasal mucosa and only limited evidence of viral presence and replication in tonsils of some pigs was found, making that only a limited amount of virus was produced capable of reaching the blood vessels. In 2-week-old piglets, however, comparable levels of viral DNA and mRNA were detected in nasal mucosa and tonsils shortly after infection with both the wild boar and NIA3 strain, indicating that this cannot be the sole explanation for the limited visceral spread of the wild boar strain. Another explanation could be that genetic differences between both strains may impact their capacity to cross the basement membrane. *Ex vivo* studies in nasal explants have shown that the viral gE and US3 proteins play a role in passage of PRV across the basement membrane barrier and a trypsin-like serine protease appears to be involved in PRV penetration of the basement membrane.^13-15^ These *ex vivo* nasal explants could provide an interesting tool to further evaluate the (possibly reduced) capacity of the wild boar PRV strains to invade the host via the nasal mucosa. Seen the importance of the viral gE protein as a virulence factor by its involvement in this and other aspects of viral infection, including virus maturation, cellular egress, intercellular spread and anterograde transport in neurons,^16-18^ the BEL24043 gE sequence was determined and compared to the wild type NIA3 gE sequence (Fig. S3). 14 nucleotide substitutions were found in the extracellular domain, most of them leading to amino acid changes. Interestingly, one amino acid deletion was observed in the cytoplasmic tail of gE of BEL24043. Since it is expected that currently unidentified mutations in the gE tail are important to promote or reduce virulence,^19^ it seems worthwhile exploring this further in the future. However, also the sequence of other viral genes should be determined in order to identify potential mutations that could contribute to the observed difference in virulence.

Next to a reduced capacity to reach lymph and blood vessels, a reduced capacity to infect endothelial or blood cells on the one hand or reduced infection and spreading capacity in the visceral organs on the other hand could also contribute to the observed reduced capacity to replicate in visceral organs. Although we cannot exclude this possibility at this time, it was previously shown that the wild boar strain and NIA3 do not differ in their capacity to replicate in primary swine kidney, lung, skin and testis cells *in vitro*.^20^

Taken together, despite the fact that it remains uncertain why the wild boar strain is less efficient in its spread to the visceral organs, this observation likely contributes to the explanation of why its virulence is lower in pigs compared to domestic strains like NIA3 and other highly pathogenic strains. This hypothesis is in line with results of previous *in vivo* studies with attenuated strains from domestic pig origin. Indeed, it was reported that the low virulent NIA1 strain was restricted in its dissemination via lymph and blood to and replication in internal organs, despite the fact that it was capable to induce encephalitis in young piglets under 4 weeks old.^21^ Similarly, the low virulent Belgian strain NS374 was capable to infect the CNS but only a restricted replication in the respiratory tract was observed and no viremia was detected.^4^

Besides differences in spread to visceral organs, the wild boar strain also displayed a reduced capacity to replicate at primary sites of infection, like tonsils and nasal mucosa, in 15-week-old pigs compared to NIA3 (Fig. S2), which correlated with a reduced spread and replication in the nervous system. Viral DNA was only found in tonsils of two pigs at the moment of euthanasia and never in nasal mucosa. Interestingly, viral DNA was also only found in the TG and pons of the same two animals that were positive in the tonsils, suggesting that the maxillary branch of the trigeminal nerve innervating the oropharyngeal tonsil is a major gateway of PRV to the neuronal cell bodies in the trigeminal ganglion in 15-week-old pigs.^22^ In line with the latter is our observation that most 15-week-old NIA3 infected pigs that were positive for viral mRNA in the TG were also positive for viral mRNA in the tonsils (Fig. S2). Importantly, viral replication in the TG of pigs infected with the wildboar PRV strain seemed efficiently suppressed based on the absence of viral mRNA detection. Similarly as for NIA3, no evidence of increased cytokine expression in the TG was found, further supporting the view that TG neurons possess multiple intrinsic and innate defense mechanisms to suppress herpesvirus replication without being detrimental for the neuron.^23^ Next to a reduced capacity of the wildboar strain to replicate in the nasal mucosa and tonsils of 15-week-old pigs, it cannot be excluded that this strain is blocked at the level of entry or transport in axons, although this does not seem to be supported by the efficient neuronal spread in 2-week-old pigs and the presence of virus in the TG of both tonsil positive 15-week-old pigs.

In 2-week-old piglets, fewer differences in replication at the primary replication sites and neuropathogenesis between the wild boar strain and NIA3 were found than in 15-week-old pigs. The wild boar strain efficiently spread to the central nervous system via both the trigeminal and the olfactory route. Both the viral replication and cytokine mRNA expression in the TG, brainstem and olfactory bulb seemed comparable to that observed after NIA3 infection, although late viral gene expression was somewhat less consistent and IE and E gene expression was not found in the olfactory bulb of all piglets. Nevertheless, morbidity and mortality were clearly reduced compared to infection with the NIA3 strain, suggesting that PRV replication in the CNS does not necessarily implicates a fatal outcome, and that it is the combination with PRV replication in peripheral organs that exhausts the piglets and leads to mortality. Interestingly, the only piglet that had to be euthanized after infection with the wild boar strain due to severe neurological symptoms showed an exuberant induction of cytokine-related mRNAs in the olfactory bulb, with an up to 500-fold increase in IFN-γ mRNA. This supports the previously formulated hypothesis that virus- and immune-related damage induced in the olfactory bulb could be an important trigger for neurological disease upon PRV infection.^8^

The inability to detect DNA of the wild boar strain in tonsils from 2-week-old piglets from 10 days p.i. onwards was unexpected seen the long lasting detection of other PRV strains in this tissue upon infection ^24–26^ and provides a further indication that the reduced virulence of this strain could in part be related to its reduced ability to replicate at this primary replication site. Also our inability to detect viral mRNA in tonsils of 2-week-old piglets infected with the NIA3 and wild boar PRV strains was unexpected and suggests that, in contrast to 15-week-old pigs, tonsils of 2-week-old pigs are less important as primary replication sites to gain access to the trigeminal ganglion.

The absence of clinical symptoms in 15-week-old pigs while neurological symptoms were observed in 2-week-old pigs confirms that age-dependent differences in disease outcome are also present after infection with the wild boar PRV isolate. This seems to be due to a more efficient neuroinvasion in 2-week-old compared to 15-week-old pigs. In line with what has been hypothesized before for age-dependent differences in (neuro) pathogenesis after NIA3 PRV infection, this is probably due to the immature development state of the immune and nervous systems in 2-week-old pigs, allowing a more extensive viral replication.^8^^,^^27-28^

In conclusion, the reduced virulence of the wild boar strain BEL24043 upon infection of domestic pigs seems due to a severely hampered spread to visceral organs in pigs of all age categories, and to a suppressed replication at primary infection sites like nasal mucosa and tonsils in older pigs. The immature development state of the immune and nervous system in 2-week old piglets seems responsible for observed age-dependent differences, and further indications were found that viral replication and the associated immune response in the olfactory bulb are related with neurological disease.

## Materials and methods

### Animals and viruses

Twenty-three 15-week-old and twenty-six 2-week-old Belgian Landrace sows were purchased from a commercial swine herd. The sows were in good condition and tested negative in serology for PRV and classical swine fever at the beginning of the experiment. The animals were housed in BSL3 facilities on slatted floors (CODA-CERVA, Machelen). Water was available ad libitum and pigs were fed once each day. The animals were randomly assigned to the control or infection group. Animal experiments were performed in accordance with the EU and Belgian regulations on animal welfare in experimentation. The protocol was approved by the joined ethical committee of CODA-CERVA and the Scientific Institute of Public Health Belgium (procedure agreement no. 121017-02).

A second passage of the Belgian wild boar isolate BEL24043 was used. This isolate was obtained from infected brain tissue of a wolf that had been fed with offal of asymptomatic wild boars shot during hunting. The wild boar strain has been genetically characterized previously and is representative for wild boar strains circulating in south-western and central Europe.^29^ It was found to be attenuated in adult pigs, but could still induce important clinical disease in young piglets.^7^

### Experimental design

In the first experiment, nine 15-week-old sows were mock infected with PBS and fourteen sows were intranasally inoculated (1 mL/nostril) with the wild boar strain BEL24043. A small nebulizer (1-mm spray opening) fixed on a syringe was used to drip the final infectious dose of 10^5^ TCID_50_/animal into the nostrils. The sows were monitored daily for clinical symptoms and rectal body temperature. Euthanasia of one pre-defined pig of the control group and two pre-defined pigs of the wild boar isolate-inoculated group was foreseen at 1, 2, 3, 5, 7, 14 and 28 days p.i. Two additional pigs of the control group were euthanized at 0 days p.i. At euthanasia, blood and several tissues (nasal mucosa, tonsils, lung, kidney, liver, spleen, olfactory bulb, TG, pons, cerebellum and cerebrum) were collected using new disposable scalpels and forceps per tissue sample to eliminate the risk of cross contamination.

In the second experiment, sixteen female 2-week-old piglets were intranasally inoculated via a small nebulizer (0.5 mL/nostril) with a final infectious dose of 10^5^ TCID_50_/animal of the wild boar isolate. Ten piglets were mock-inoculated with PBS and held as a negative control group. Piglets were monitored daily for clinical symptoms and rectal body temperature. Based on the low virulence observed in 15-week-old pigs, euthanasia of one pre-defined pig of the control group and two pre-defined pigs of the wild boar isolate-inoculated group was foreseen at 1, 2, 3, 4, 5, 7, 14 and 21 days p.i. Two additional pigs of the control group were euthanized at 0 days p.i. During the experiment, animals were euthanized at the intended date or when necessary based on ethical grounds. One piglet had to be euthanized on ethical grounds at 5 days p.i. instead of at 21 days p.i. as foreseen. At that moment, it was decided to euthanize one of the 3 remaining piglet at 10, 14 and 21 days p.i., respectively. Same samples as for 15-week-old pigs were collected at the moment of euthanasia.

### Sample preparation and DNA extraction

For all collected organs, except tonsils, about 0.5 cm^3^ of tissue was homogenized in 1 mL phosphate buffered saline (PBS) by adding 10–15 silicon carbide beads of 1 mm (Biospec Products Inc.) and high speed shaking (4 min, 25 Hz) in a TissueLyser (Qiagen). For homogenization of tonsil samples, about 0.5 cm^3^ of tissue was homogenized in 1 mL PBS by adding 2 stainless steel beads of 5 mm (Qiagen) and high speed shaking (10 min, 30 Hz) in a TissueLyser. For the 2-week-old piglets, homogenization of samples was done in only 0.5 mL of PBS because of the limited amount of sample available. Genomic DNA was extracted from the homogenized tissues using the QiAmp DNA kit (Qiagen) following manufacturer's instructions.

### RNA extraction and cDNA synthesis

Extraction of total RNA from the homogenized tissue preparations was done by the RNeasy Mini kit (Qiagen) following manufacturer's instructions. Extracted total RNA was treated with Turbo DNase (Ambion) following manufacturer's instructions in a 50 µL reaction to eliminate contaminating DNA. Subsequently DNase treated RNA samples were converted to cDNA using the M-MLV reverse transcriptase system (Life Technologies). For each reaction, a mix of 4 μl RNA, 4 μl 5 × first strand buffer, 2 μl 0.1 M DTT, 1 μl 10 nM dNTP mix (Roche, Basel, Switzerland), 0.2 μl 10 × hexanucleotide mix (Roche, Basel, Switzerland), 0.5 μl M-MLV RT and 8.3 μl H_2_O was prepared and incubated at 37°C for 60 min, followed by inactivation at 95°C for 10 min. Due to overlapping open reading frames of the large latency transcript (LLT) and IE180 and EP0 on cDNA strands, a separate reverse transcription was performed for downstream detection of IE180 and EP0 whereby the hexanucleotide mix was replaced by 1 µl containing 10 µM of IE180 and EP0 reverse primers.

### Preamplification

A preamplification step of the cDNA was done using the TaqMan PreAmp Master Mix (Applied Biosystems) following manufacturer's instructions to increase the amount of available material. A mix of primers for the selected reference genes, cytokines and viral genes (except IE180 and EP0) was made to a final concentration of 0.18 µM per primer.^8^ Also a separate mix containing only the IE180 and EP0 primers was made to preamplify the cDNA produced using the IE180 and EP0 reverse primers (see above). Preamplification reaction conditions involved the amplification of 12.5 μl cDNA in a 50 μl reaction consisting of 25 μl TaqMan PreAmp Master Mix and 12.5 μl pooled assay mix. Pre-amplification consisted of 14 cycles on a Thermocycler (Biometra) with the following program: denaturation at 95°C for 10 min and 14 cycles of amplification (15 sec at 95°C, 4 min at 60°C). The preamplified products were diluted at a ratio 1:10 and used as templates for the real-time qPCR analysis. The preamplification uniformity of the different target sequences in nasal mucosa, trigeminal ganglia, pons and olfactory bulb had been confirmed in a previous study.^8^ The mean PreAmp uniformity values for the investigated gene assays in the tonsils related to the reference genes GADPH, ACTB and HPRT1 were -0.17 ± 1.06, -0.72 ± 1.06 and 0.03 ± 1.06, respectively. PreAmp uniformity values between -1.5 and +1.5 are considered as acceptable.

### Quantitative real-time PCR (qPCR) assay design and analysis

Previously published primers and probes for seven porcine reference genes (ACTB, B2M, GADPH, HPRT1, PPIA, SDHA and UBC), eleven cytokines (IFN-α, IFN-β, IFN-γ, TNF-α, IL1α, IL1β, IL2, IL4, IL6, IL8 and IL10) and six PRV genes (IE180, EP0, gB, gC, gE, LAT intron) were used.^8^ All probes were labeled with 6-Carboxyfluorecein (FAM). The real-time PCR reactions were performed in a final volume of 20 μl, containing 5 μl DNA or cDNA, 10 μl FastStart TaqMan Probe Master Mix 2x (Roche), 1 μl probe (final concentration 0.25 μM), 1 μl of a mix containing the forward and reverse primer (both primers at a final concentration 0.9 μM) and 3 µL H_2_O. The following real-time RT-PCR temperature cycle was applied: denaturation by a hot start at 95°C for 10 min, followed by 45 cycles of denaturation at 95°C for 15 sec and annealing/extension at 60°C for 45 sec. All experiments were performed in duplicate on a LightCycler 480 Real-Time PCR system (Roche, Basel, Switzerland). In all qPCR analyses performed, negative extraction controls and negative and positive amplification controls were included and tests were only validated when all controls were satisfactory. For the interpretation of the viral gene expression, it should be kept in mind that the qPCRs for IE180 and EP0 have a 100-fold higher limit of detection than those for the viral glycoproteins.^8^

### Construction of a standard curve for qPCR for the glycoprotein B (gB) assay

A gB DNA standard curve was constructed for absolute quantification of viral DNA present in the different tissues. A plasmid containing part of the gB sequence was 10-fold serially diluted in a DNA extract from a homogenate of PRV negative organs resulting in a dilution series from 5 × 10^9^ to 5 × 10^−1^ copies/μl. All dilutions were tested in the qPCR described above. Three independent replicates were run, mean values of each dilution were calculated and a standard curve was constructed by plotting the Ct values against the log of the input DNA copy number.

### geNorm analysis and relative quantification

As previously determined and described,^8^ following reference genes were used for normalization of qPCR data: UBC and GADPH for nasal mucosa samples, ACTB, GADPH and PPIA for TG samples, and UBC and PPIA for CNS samples. Here we determined the reference genes necessary for normalization in tonsils. Based on the geNorm analysis as described before,^8^ GADPH, ACTB and HPRT1 were selected as reference genes for tonsils.

The quantification and normalization of results were based on the calculation of target Ct values and reference gene Ct values in qBasePlus software. All samples were run in duplicate for the target and reference genes. Relative expression levels were normalized with respect to the selected reference genes, and to technical and experimental errors. Relative expression quantification analysis relied on the qBase method.^30^ Individual cytokine levels for all animals were expressed relative to the average of the control group (separately for 2- and 15- week old animals). Viral gene levels were expressed relative to the lowest positive sample for each age category.

### Enzyme-linked immunosorbent assay (ELISA)

Serum samples collected at the day of euthanasia were tested by an ELISA detecting antibodies directed against the gB glycoprotein (PrioCHECK PRV gB Antibody ELISA kit; Prionics) following the manufacturer's instructions. For each sample, the sample/positive (S/P) percentage was calculated. Test results were considered negative if S/P < 50% and positive if S/P > 50%.

## Supplementary Material

KVIR_S_1368941.zip
