# Supplementary material for: Reduced virulence of a pseudorabies virus isolate from wild boar origin in domestic pigs correlates with hampered visceral spread and age-dependent reduced neuroinvasive capacity
Source: Virulence. 2017 Oct 4;9(1):149–62. doi: 10.1080/21505594.2017.1368941 (PMC5955469; doi:10.1080/21505594.2017.1368941)
Supplement: KVIR_S_1368941.zip [file kvir-09-01-1368941-s001.zip › KVIR_S_1368941.docx]

**Supplemental figure 1.**


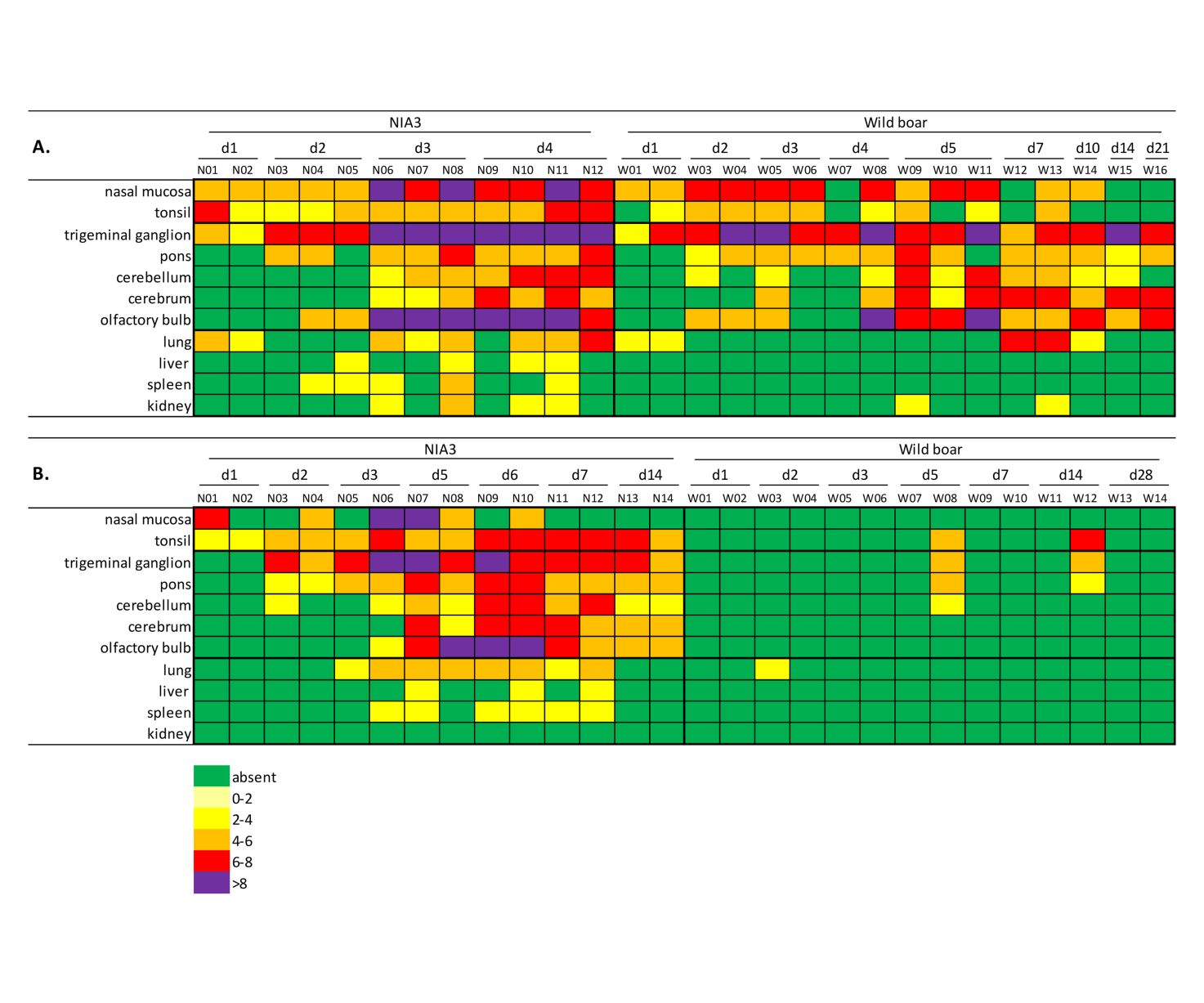


Distribution of viral DNA in different tissues of domestic pigs infected with PRV strain NIA3 or BEL24043. Several tissues were collected from 2-week-old (panel A) and 15-week-old (panel B) pigs at different time points after intranasal inoculation with 10^5^ TCID_50_ of the NIA3 reference strain (domestic pig origin) or the BEL24043 strain (wildboar origin) and tested by qPCR directed against glycoprotein gB. Viral DNA concentrations found in different tissues are shown. Color codes indicate ranges of log_10_ copies per gram tissue.

**Supplemental figure 2.**


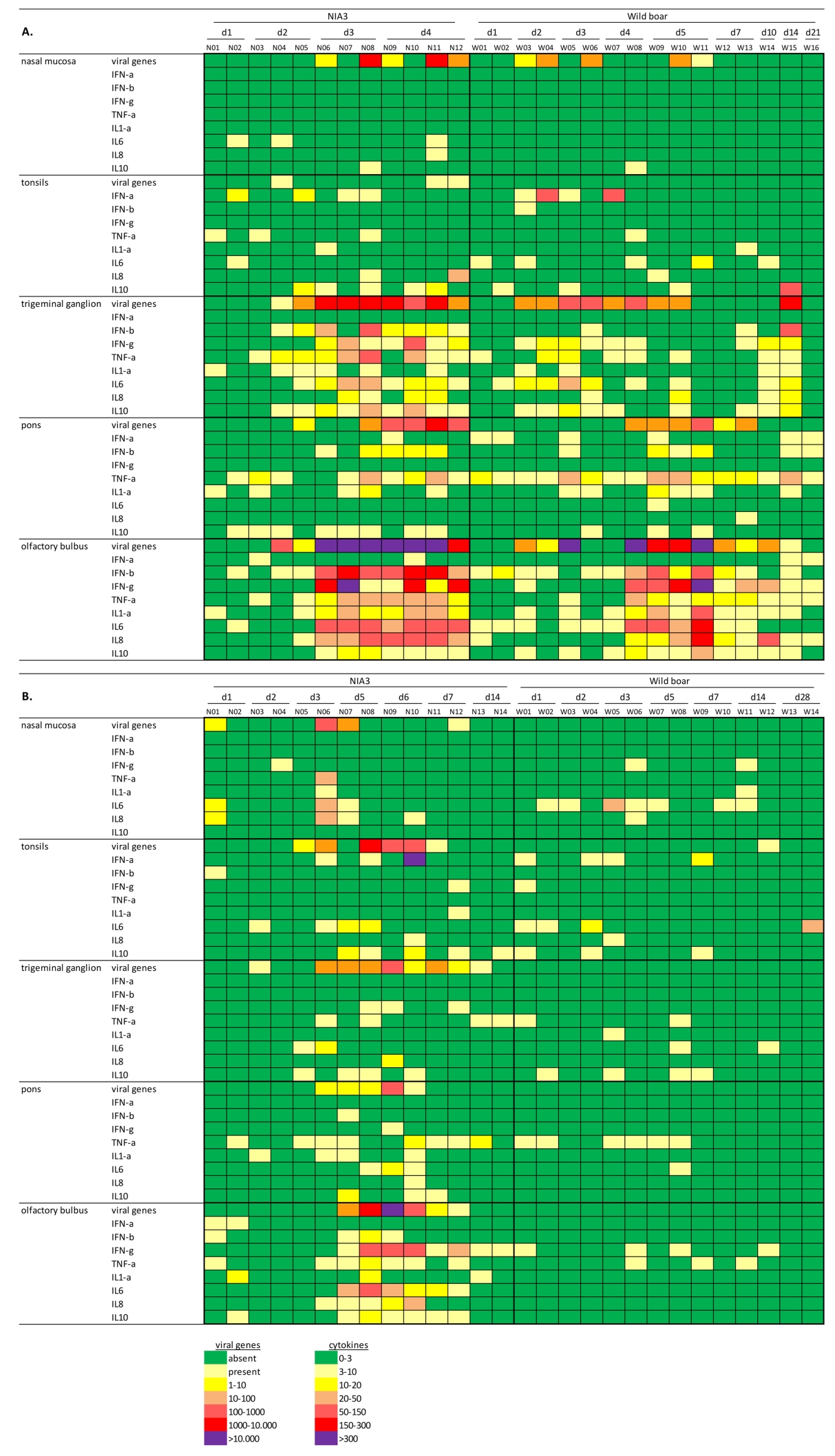


Changes in viral gene and cytokine mRNA expression in different tissues of domestic pigs infected with PRV strain NIA3 or BEL24043. Domestic pigs of 2-week-old (panel A) and 15-weeks-old (panel B) were intranasally inoculated with 10^5^ TCID_50_ of the NIA3 strain (domestic pig origin) or the BEL24043 strain (wild boar origin) and euthanized at different time points post infection. Viral and cytokine mRNA expression in different tissues was studied by RT-qPCR. The colour code for the viral genes indicates the maximum increase relative to the lowest positive sample for each strain and pig age category observed among the 6 viral genes (IE180, EP0, gB, gE, gC, LAT) that were studied. The colour code for the cytokine-related mRNA expression indicates the fold change of each cytokine under study expressed relative to the average cytokine expression in the control group that was included for each strain and pig age category.

**Supplemental figure 3.**

| nucleotide | | | | | amino acid | | | | |
| --- | --- | --- | --- | --- | --- | --- | --- | --- | --- |
| NIA3 | |  | BEL24043 | | NIA3 | |  | BEL24043 | |
| 103 | g | **—** | **›** | a | 35 | V | **—** | **›** | T |
| 113 | t | **—** | **›** | c | 38 | V | **—** | **›** | A |
| 114 | c | **—** | **›** | g |  |  |  |  |  |
| 163 | a | **—** | **›** | g | 55 | N | **—** | **›** | D |
| 221 | t | **—** | **›** | g | 74 | L | **—** | **›** | R |
| 371 | t | **—** | **›** | c | 124 | M | **—** | **›** | T |
| 427 | c | **—** | **›** | g | 143 | P | **—** | **›** | A |
| 432 | g | **—** | **›** | t | 144 | E | **—** | **›** | D |
| 485 | a | **—** | **›** | g | 162 | Q | **—** | **›** | R |
| 506 | c | **—** | **›** | t | 169 | T | **—** | **›** | I |
| 539 | g | **—** | **›** | a | 180 | R | **—** | **›** | Q |
| 588 | g | **—** | **›** | a |  |  |  |  |  |
| 685 | t | **—** | **›** | c | 229 | F | **—** | **›** | L |
| 1047 | c | **—** | **›** | t |  |  |  |  |  |
| 1482 | c | **—** | **›** | / | 494 | D | **—** | **›** | / |
| 1483 | g | **—** | **›** | / |  |  |  |  |  |
| 1484 | a | **—** | **›** | / |  |  |  |  |  |

Detailed overview of DNA and amino acid substitutions in the gE gene of BEL24043 compared to NIA3. The gE sequence was obtained via sanger sequencing of 5 overlapping PCR fragments overspanning the gE gene of a second passage isolate of the BEL24043 strain and submitted to Genbank (MF188844). Nucleotide (nt) and amino acid (aa) positions are predicted based on the gE gene sequence of the NIA3 strain (GenBank accession nr. KU900059; Mathijs et al., 2016).
